# The association between social support provision, psychological capital, subjective well-being and sense of indebtedness among undergraduates with low socioeconomic status

**DOI:** 10.1186/s40359-023-01325-w

**Published:** 2023-09-27

**Authors:** Zhongyi Xin

**Affiliations:** Faculty of Education Science, Shaanxi Xue Qian Normal University, No.101Shenhe 2Nd Road, Chang An District, Xi’an, 710100 China

**Keywords:** Social support provision, Psychological capital, Subjective well-being, Sense of indebtedness

## Abstract

**Background:**

Social support consists of receipt and provision in the interpersonal exchange process. Many studies have explored and verified the effect of received social support. This study focuses on whether and when social support provision can benefit the providers’ positive psychological capital and subjective well-being.

**Methods:**

A sample of 732 Chinese undergraduates with low socioeconomic status completed questionnaires on social support provision, psychological capital, life satisfaction, positive affect, negative affect, and sense of indebtedness.

**Results:**

The correlation and regression analyses showed that impoverished college students’ social support provision was positively associated with life satisfaction, positive affect, and psychological capital and negatively associated with negative affect. The interaction between the sense of indebtedness and social support provision was negatively associated with life satisfaction, positive affect, and psychological capital, not significantly associated with negative affect.

**Conclusion:**

The results demonstrated that giving social support can be as beneficial as receiving social support, and the sense of indebtedness can limit the benefits. Individuals with a lower sense of indebtedness are more likely to benefit from social support provision. The findings have implications for marginalized groups’ subjective well-being and positive psychological capital and show the necessity of guiding individuals to provide social support while maintaining their autonomy.

## Introduction

Social support consists of receipt and provision in the interpersonal exchange process [1]. Many studies have explored and verified the effect of social support that is received (or perceived), such as health [2], positive feelings [3], mental health [4], engagement [5], well-being [6], the positive effect of social support provision is not fully explored. Existing studies have mainly focused on the impact of the elderly’s social support provision on physical health [7] and longevity [8], and less attention has been paid to the positive impact of social support provision on youth groups, such as psychological capital and subjective well-being. As a key psychological resource, psychological capital significantly influences individuals’ development [9]. Subjective well-being is an essential developmental indicator of the individual, comprising long-term levels of positive affect, life satisfaction, and lack of negative affect [10].

The personal well-being model suggests that giving behaviors can enhance and improve self-evaluation and build self-psychological resources [11]. It is reasonable to infer that giving social support may increase the individual’s psychological capital. According to the need to belong theory, interpersonal interaction is an essential source of subjective well-being [12]. Social support provision contains many deep interpersonal interactions and may profoundly influence subjective well-being. More importantly, the buffer role for the psychological capital and subjective well-being of an individual with low socioeconomic status, recognized as more generous in supporting others [13], should be explored. However, insufficient economic resources may deteriorate subjective well-being [14] and psychological capital [15]. It is of theoretical and practical importance to explore the potential influence of social support provision on impoverished individuals' psychological capital and subjective well-being.

The sense of indebtedness is an emotion produced by an individual after receiving the favor of others, and different individuals have different levels of indebtedness after receiving the same favor. Compared to individuals with a low sense of indebtedness, individuals with a high sense of indebtedness are more likely to experience indebtedness and thus have a stronger sense of obligation and compulsion to repay [16]. Every year in China, many impoverished college students are guided to give back after receiving a national bursary by doing things beneficial to others and society. It is unknown whether this situation will induce a higher sense of indebtedness in individuals with high indebtedness and affect the autonomy of social support provision. Giving social support is a self-initiated and self-determined behavior. If giving is affected by a sense of obligation, will it reduce the positive impact of this autonomous behavior? To fill this gap, this study focused on the link between social support provision, psychological capital and subjective well-being in college students with different sense of indebtedness.

### Social support provision and subjective well-being

Social support involves receiving and providing in any process of interpersonal exchange. The former refers to perceived or received care and respect from others [17], while the latter refers to providing care and respect to others [18]. The two influence each other in interpersonal interaction. People may actively provide social support to others or provide emotional or instrumental support to others after receiving support from them [19]. However, social support in psychology research is tacitly regarded as received or perceived care from others. Many scholars have researched the influence of social support on recipients, but the positive impact of social support on the provider has yet to be emphasized. A few studies have confirmed the positive effect of social support provision on the providers’ physical health and mortality [20, 21]. However, not enough studies have focused on the psychological benefit of social support provision on the provider, especially on their subjective well-being.

Providing social support to others is prosocial behavior. From the perspective of biological evolution, providing support to others is an instinctive behavior with adaptive significance. According to the self-motivation effect of altruism in the evolutionary process [22], giving emotional support can enable helpers to adjust their psychological resources to engender positive influence. Therefore, from an evolutionary point of view, providing emotional support, such as comfort to others, can also help individuals obtain psychological benefits. The increase in positive emotions, reduction in negative emotions, and improvement in life satisfaction are likely important results of social support provision. In addition, social exchange theory believes that human behavior is dominated by exchange activities that can bring rewards and follow the principle of reciprocity and equality; providing emotional support to others can improve their emotions and create positive emotions and life satisfaction [23].

From a psychological point of view, compared with receiving social support, the behavior of giving social support is an autonomous behavior, which itself has the effect of enhancing well-being. The model of longitudinal well-being posited that intentional engagement could trigger increasing and lasting well-being than life circumstances [24]. In other words, individuals can obtain more happiness through activities toward others as a “positive happiness maker” but not as a “negative owner.” Moreover, the interdependence theory suggests that individuals’ well-being is independent of the satisfaction of their own needs but dependent on the satisfaction of social needs, attributable to the establishment, maintenance, and enhancement of interpersonal relationships [25]. Providing social support to others refers to establishing deep interpersonal connections; thus, it is reasonable to infer that it could positively impact subjective well-being. Previous research has found that prosocial behaviors can significantly and positively predict life satisfaction and positive emotions [26] and negatively predict negative emotions [27]. Social support provision is one type of giving behavior. Therefore, this study hypothesized that social support provision could significantly predict individuals’ subjective well-being.

Research on the relationship between social support and the life satisfaction of the elderly found that social support can promote the elderly’s life satisfaction, especially for the elderly over 75 years of age, Because the elderly is more easily seen as weak and incompetent when the elderly provides social support to others, it can break stereotypes, enhance self-esteem, and thus have a positive effect on the individual [28]. Similarly, college students with low economic status are regarded as vulnerable groups and individuals who need help, and their positive giving behavior may also play a role in improving individual well-being. Based on this, this study assumes that the social support provision of college students with low socioeconomic status correlates significantly with subjective well-being.

### Social support provision and psychological capital

The personal well-being model suggests that giving behaviors can enhance and improve self-evaluation and build self-psychological resources. However, the giving may be initiated by different goals and motivations [29]. Providing emotional support to others to overcome difficulties can help accumulate experience in dealing with stressful events and increase self-evaluations and perceived competence [30], mental resilience, and sense of value [31]. On the other hand, it can distract help-givers from focusing on their troubles and stress [32] and facilitates psychological adaption via the shift of internal standards, values, and the conceptualization of well-being [33]. After social support is given, individuals may be more resilient in the face of difficulties, have a more positive self-evaluation, have a more optimistic attitude towards success, and be full of hope for the future [34]. This state is consistent with the concept of psychological capital, defined as a positive psychological state composed of self-efficacy, hope, optimism, and resilience [35]. Individuals with high psychological capital rate success more positively and perceive themselves as possessing sense of control and resilience to setbacks [36]. In short, the provision of care to others increases psychological capital. Thus, it can be inferred that social support provision may promotes individuals’ psychological capital.

### Sense of indebtedness as a moderator

Although studies have confirmed the positive effect of social support provision, some studies have found that giving does not positively impact the giver [37]; giving to others may sometimes burden the provider. A study on family caregivers finds that more caregiving was linked to lower life satisfaction [38]. It can be seen that there are certain conditions for giving behaviors to produce benefits. As mentioned earlier, the self-determination theory emphasizes that the autonomy of individual behavior is an essential condition for behaviors to have positive effects. This means that behaviors must be voluntary and freely chosen [39]. Additionally, according to the hypothesis proposed by Inagaki and Orehek [40], freedom of giving is necessary for social support provision to generate benefits. Existing studies have also found that being forced to participate in prosocial behaviors may be counterproductive and have adverse effects [41].

A sense of indebtedness is the obligation to repay another, which is often a relatively stable and lasting emotional state after receiving support from others and society [42, 43]. Different individuals differ significantly in their sense of indebtedness after receiving [44, 45]. The greater the state of indebtedness, the greater the pressure to compel individuals to provide feedback to others [46] to seek a balance in social interaction and achieve the goal of mutual benefit, equality, and fairness [47]. The level of the sense of indebtedness may vary after receiving a national bursary, so there must be significant differences in the sense of pressure to repay, and there are also great differences in the level of autonomy of social support given. For college students with a high sense of indebtedness, the autonomy of giving behaviors decreases and turns into stressful behaviors, so the benefits of giving behaviors may decrease or even disappear. However, college students with low indebtedness also receive the same national bursary. They have a lower pressure to repay, so they have a higher degree of autonomy in giving behavior, and their giving behavior can still produce benefits. From this point of view, although the individual initiates social support, the benefit of giving may be affected by the sense of indebtedness. Whether the degree of the sense of indebtedness, which is induced by receiving state financial aid, could limit the benefits generated by social support provision needs to be further explored.

### The current study

This study aims to examine the association between social support provision, sense of indebtedness, psychological capital, and subjective well-being of undergraduates with low socioeconomic status. The regression model was employed to interpret the following questions: (a) Can social support provision be positively associated with psychological capital and subjective well-being? (b) can sense of indebtedness weaken the benefit of social support provision?

## Materials and methods

### Participants

The college students who received state financial aid were taken as the participants. College students with an annual family income of less than 3,000 yuan can apply for state financial aid. Students apply to their department in the first month of enrollment, and the student management office verifies the application. After the verification is passed, it will be included in the school’s poor student database. The database covers all impoverished college students receiving financial aid. The students selected for this study are from this database.

The researcher sent survey requests to the student management offices of 6 colleges and universities in the Midwest, and four responded to our request. The researcher informs student administrators of respondent eligibility criteria to ensure that the number of students in each grade and gender is relatively balanced. After finalizing the list of participants, the student management office sent survey requests to the assistant of each faculty and got approval from them. Then the researchers conducted investigation training through online meetings. The assistant investigated at the weekly class meeting. The teacher first explained the requirements of the investigation, provided informed consent, and emphasized the voluntary nature and anonymity of the investigation. After scanning the QR code, students fill out the questionnaire and get 2-yuan or 20-yuan coupon rewards. Due to the use of an online platform (Questionnaire Star), the platform required that all questions be answered before submitting or dropping out. No students dropped out of the survey. The researcher aggregates the data collected.

A total of 800 questionnaires were returned, and 68 were excluded because they did not meet the selection criteria, which provided the incorrect answer to the following question: “What is the capital of China” (Beijing). This question was included to determine whether the participants had taken the questionnaire randomly. The chi-square test results show that there is no significant difference in gender (χ^2^(1,800) = 0.29,* p* > 0.05) and age (χ^2^(5,800) = 0.41, *p* > 0.05) between the excluded participants and the final sample, indicating that there is no structural missing. Finally, 732 valid questionnaires were collected: 336 (45.9%) participants were male, and 396 (54.1%) were female; 203 first-year students, 196 sophomores, 176 juniors, and 157 seniors; and their ages ranged from 18 to 23.

## Measures

### Social support provision questionnaire

The emotional social support provision scale [48] was adopted in this study. The confirmatory factor analysis was performed for the Chinese revision, and the model fit well [49]. The scale has three items, each rated on a 5-point Likert scale (1 = never, 5 = always). A higher score indicates more emotional social support provided to others. Cronbach’s α of the emotional social support provision scale in the present study was 0.81.

### Sense of indebtedness questionnaire

Sense of indebtedness was measured by the Indebtedness Questionnaire [16, 50]. The result of CFA for the Chinese version fits well: χ^2^/*df* = 4.932, GFI = 0.951, TLI = 0.934, CFI = 0.961, RMSEA = 0.076, SRMR = 0.035. The scale consisted of five items. The scale was rated on a 5-point Likert scale (1 = strongly disagree, 5 = strongly agree). The higher the total score, the greater the sense of indebtedness a person feels. The Cronbach’s α of the Indebtedness Questionnaire in the present study was 0.86.

### Psychological capital questionnaire

Psychological capital was measured by the Psychological Capital Questionnaire Chinese revision [51]. The model fit index in this study meets the requirements: χ^2^/*df* = 3.899, GFI = 0.915, TLI = 0.956, CFI = 0.985, RMSEA = 0.043, and SRMR = 0.032. The scale consists of 26 items, including four dimensions: self-efficacy, resilience, optimism, and hope. Each item was rated on a 5-point Likert scale (1 = never, 5 = always). The total score of psychological capital reflects the individual’s psychological capital status. The higher the score, the higher the level of positive psychological capital. The Cronbach’s α of the Psychological Capital Questionnaire in the present study was 0.91.

### Subjective well-being questionnaire

Based on the theory of subjective well-being, Individuals with more life satisfaction and positive affect and less negative affect are happier [52]. Life satisfaction was measured by the satisfaction with life scale [52]. The Chinese version of life satisfaction was revised and validated by Xiong & Xu [53]. The model fits well in this study: χ^2^/*df* = 4.389, GFI = 0.978, TLI = 0.928, CFI = 0.964, RMSEA = 0.054, SRMR = 0.037. The 5-item scale was rated on a 5-point Likert scale (1 = strongly disagree, 5 = strongly agree), and the total score indicates the level of life satisfaction. The Cronbach’s α of life satisfaction in the present study was 0.81.

The Chinese version of the affect balance scale [54] was used to measure positive and negative affect. Six items were used to assess positive affect (Cronbach’s α = 0.84), and six were used to assess negative affect (Cronbach’s α = 0.84). The model fit of negative affect is as follows: χ^2^/*df* = 3.469, GFI = 0.965, TLI = 0.974, CFI = 0.985, RMSEA = 0.061, SMRR = 0.0252. The model fit of positive affect is as follows: χ^2^/*df* = 5.94, GFI = 0.975, TLI = 0.947, CFI = 0.968, RMSEA = 0.086, SRMR = 0.041. Each item was rated on a 5-point Likert scale (1 = never, 5 = always), and positive and negative affect were computed. A higher score on each scale means more positive/negative affect.

### Procedure

This study was conducted in 2021 at four universities in Xi’an, Taiyuan, and Wuhan. Ethical approval was obtained from the researcher’s university ethics committee. Self-report questionnaires were completed after obtaining informed consent. Participants filled out questionnaires regarding the social support provision, sense of indebtedness, psychological capital, and subjective well-being. It took 10–15 min to complete the questionnaires. The participants were free to withdraw from the study at any time. They did not place their names on the measures, and the confidentiality of their responses was assured.

### Data analysis

First, the descriptive analysis of observed variables was performed by IBM SPSS Statistics 25. Second, a multiple regression model was conducted by Mplus 8.3 to determine the association between social support provision, psychological capital, and subjective well-being. In this analysis process, the research variables are converted into latent variables before incorporating them and control variables into the model. Third, the interaction between social support provision and the sense of indebtedness was entered into the regression model to test the moderated effect of the sense of indebtedness.

## Results

### Common method variance test

The pre-procedure and post-statistics were used to control and test the possible common method variance. The study found that the tendency to pursue consistent responses is one of the sources of common method variance [55]. Therefore, different response words were used in the questionnaires. Some questionnaires used 1 = strongly disagree, 5 = strongly agree, and some used 1 = never, 5 = always. To eliminate the concerns of the participants, all scales were conducted anonymously. The collection of demographic information was placed at the end of the questionnaire. The *Harman* single-factor test was performed to evaluate the common method variance. There were nine factors with eigenvalues greater than 1, and the variance explanation rate of the first factor was 29.906%, which was less than the recommended value of 40% [55]. Using *Mplus* 8.3 to perform confirmatory factor analysis (CFA) on the single-factor model, the results showed that the model fitting index did not meet the requirements ($\chi$ ^2^/*df* = 5.449, *CFI* = 0.481, *TLI* = 0.473, *RMSEA* = 0.173, *SRMR* = 0.132). Moreover, the unmeasured latent method factor (ULMC) was used to further test for common method variance. First, construct a model loading the items on their corresponding constructs (model1:χ^2^/*df* = 3.345*, CFI* = 0.918, *TLI* = 0.901, *RMSEA* = 0.059, *SRMR* = 0.064). Second, added a method factor to the model1(model2: χ^2^/*df* = 3.273*, CFI* = 0.926, *TLI* = 0.919, *RMSEA* = 0.047, *SRMR* = 0.051). Third, compared Model 2 with Model 1. The fit of model2 did not significantly improve compared to model1(ΔCFI = 0.008, ΔTLI = 0.018, ΔRMSEA = 0.012, ΔSRMR = 0.013). The above method has reduced the common method variance to a certain extent.

### Descriptive statistics and correlations

Table 1 shows the mean, standard deviation, and correlation of the variables. The correlation analysis revealed that social support provision was positively related to psychological capital (*r* = 0.344, *p* < 0.01), life satisfaction (*r* = 0.293, *p* < 0.01), and positive affect (*r* = 0.348, *p* < 0.01); psychological capital is positively related to life satisfaction (*r* = 0.536, *p* < 0.001) and positive affect (*r* = 0.607, *p* < 0.001); social support provision is negatively related to negative affect (*r* =  − 0.13, *p* < 0.001); psychological capital is negatively correlated with negative affect (*r* =  − 0.243, *p* < 0.01) and sense of indebtedness (*r* =  − 0.141, *p* < 0.01); and the sense of indebtedness is positively correlated with negative affect (*r* = 0.247, *p* < 0.01).

**Table 1:** Descriptive statistics and correlations

Variables	M ± SD	SSP	PSYCAP	LS	PA	NA	INDEB
SSP	3.542 ± 0.513	-					
PSYCAP	3.34 ± 0.353	0.344^**^	-				
LS	3.058 ± 0.649	0.293^**^	0.536^***^	-			
PA	3.696 ± 0.503	0.348^**^	0.607^***^	0.633^***^	-		
NA	2.686 ± 0.639	-0.130^**^	-0.243^**^	-0.280^**^	-0.416^**^	-	
INDEB	3.673 ± 0.585	0.094	-0.141^**^	-0.022	0.071	0.247^**^	-

*SSP* social support provision; *PSYCAP* psychological capital; *LS* life satisfaction; *PA* positive affect; *NA* negative affect; *INDEB* sense of indebtedness

^*^p < 0.05

^**^p < 0.01

^***^p < 0.001

### Testing for the effect of social support provision on psychological capital and subjective well-being

To examine the effect of social support provision on psychological capital and subjective well-being, the regression model was tested by using *Mplus* 8.3. First, take age and gender as the predictors, psychological capital, positive affect, negative affect, and life satisfaction as outcomes. The relationship between gender, age, and the outcome variables was insignificant. Second, take age and gender, social support provision, and sense of indebtedness as the predictors, psychological capital, positive affect, negative affect, and life satisfaction as outcomes. The model fitted the data well (χ^2^/*df* = 2.665, CFI = 0.928, TIL = 0.916, RMSEA = 0.05, SRMR = 0.044).The result showed that the social support provision was positively associated with psychological capital(*b* = 0.466, *t* = 11.418, *p* < 0.001, Cohen’s ƒ^2^ = 0.286), life satisfaction(*b* = 0.317, *t* = 6.607, *p* < 0.001, Cohen’s ƒ^2^ = 0.114) and positive affect(*b* = 0.388, *t* = 9.481, *p* < 0 0.001, Cohen’s ƒ^2^ = 0.191), negatively associated with negative affect(*b* = -0.173, *t* = 3.748, *p* < 0.01, Cohen’s ƒ^2^ = 0.173), The results are presented in Table 2.

**Table 2:** The effect of social support provision

outcomes	predictors	*b*	*t*	*LLCI*	*ULCI*
PSYCAP	gender	-0.033	-0.768	-0.117	0.051
	age	0.037	0.863	-0.047	0.121
	SSP	0.466	11.418^***^	0.386	0.546
	INDEB	0.051	1.129	-0.037	0.139
LS	gender	-0.021	-0.461	-0.109	0.068
	age	-0.034	-0.721	-0.126	0.058
	SSP	0.317	6.607^***^	0.223	0.411
	INDEB	-0.003	-0.064	-0.104	0.097
PA	gender	0.024	0.611	-0.053	0.101
	age	0.024	0.524	-0.066	0.114
	SSP	0.388	9.481^***^	0.308	0.468
	INDEB	-0.001	-0.028	-0. 094	0.092
NA	gender	-0.015	-0.333	-0.101	0.072
	age	-0.033	-0.721	-0.122	0.056
	SSP	-0.173	-3.748^**^	-0.264	-0.083
	INDEB	0.295	6.517^***^	0.206	0.383

*SSP* social support provision, *PSYCAP* psychological capital, *LS* life satisfaction, *PA* positive affect, *NA* negative affect, *INDEB* sense of indebtedness

^**^p < 0.01

^***^p < 0.001

### Testing for the moderated effect of sense of indebtedness

The study assumes that sense of indebtedness weakens the association between social support provision, psychological capital and subjective well-being. To verify the moderating effect of sense of indebtedness, take the age, gender, social support provision, sense of indebtedness, and the interaction between social support provision and the sense of indebtedness as predictors, psychological capital, positive affect, negative affect, life satisfaction as outcomes. The regression result found that the interaction between social support provision and the sense of indebtedness was significantly associated with psychological capital(*b* = -0.091, *t* = -2.144, *p* < 0.05, Cohen’s ƒ^2^ = 0.03), life satisfaction(*b* = -0.103, *t* = -2.165, *p* < 0.05, Cohen’s ƒ^2^ = 0.08), positive affect(*b* = -0.142, *t* = -3.078, *p* < 0.01, Cohen’s ƒ^2^ = 0.119), but not significantly associated with the negative affect(*b* = -0.005, *t* = 0.115, *p* > 0.05, Cohen’s ƒ^2^ = 0.01). The details are presented in Table 3.

**Table 3:** The moderated effect of sense of indebtedness

outcomes	predictors	*b*	*t*	*LLCI*	*ULCI*
PSYCAP	gender	-0.037	-0.972	-0.37	0.122
	age	0.043	1.123	-0.036	0.114
	SSP	0.452	11.49^***^	0.376	0.531
	INDEB	0.058	1.327	-0.046	0.124
	INT	-0.091	-2.144^*^	-0.181	-0.015
LS	gender	-0.025	-0.604	-0.338	0.197
	age	-0.028	-0.663	-0.11	0.053
	SSP	0.303	6.66^***^	0.212	0.390
	INDEB	-0.002	-0.006	-0.114	0.07
	INT	-0.103	-2.165^*^	-0.187	-0.004
PA	gender	0.017	0.438	-0.217	0.29
	age	0.032	0.818	-0.052	0.103
	SSP	0.371	8.654^***^	0.298	0.465
	INDEB	0.006	0.136	-0.074	0.102
	INT	-0.142	-3.078^**^	-0.231	-0.053
NA	gender	-0.014	-0.352	-0.266	0.246
	age	-0.033	-0.82	-0.109	0.048
	SSP	-0.17	-3.783^***^	-0.266	-0.09
	INDEB	0.294	6.752^***^	0.219	0.388
	INT	0.005	0.115	-0.096	0.095

*SSP* social support provision, *PSYCAP* psychological capital, *LS* life satisfaction, *PA* positive affect, *NA* negative affect, *INDEB* sense of indebtedness, *INT* the interaction between social support provision and sense of indebtedness

^*^p < 0.05

^**^p < 0.01

^***^p < 0.001

To clarify the essence of the interaction between social support provision and the sense of indebtedness, following Aiken [56] and Hayes’s [57] recommendation, the average level of indebtedness + 1SD is defined as a high level of indebtedness and—1SD as a low level of indebtedness. As shown in Table 4, compared with individuals with high level of indebtedness, social support provision was associated with greater psychological capital, life satisfaction, and positive affect in individuals with low level of indebtedness. However, there is no significant difference in the association between social support provision and negative affect under different levels of sense of indebtedness.

**Table 4:** The moderated effect of indebtedness

outcomes	level of indebtedness	simple effect	LLCI	ULCI
PSYCAP	Low	0.399	0.299	0.499
	High	0.265	0.167	0.363
LS	Low	0.41	0.271	0.55
	High	0.203	0.063	0.343
PA	Low	0.541	0.4	0.681
	High	0.241	0.099	0.384
NA	Low	-0.201	-0.342	-0.06
	High	-0.189	-0.342	-0.036

*PSYCAP* psychological capital, *LS* life satisfaction, *PA* positive affect, *NA* negative affect

**Fig. 1:**
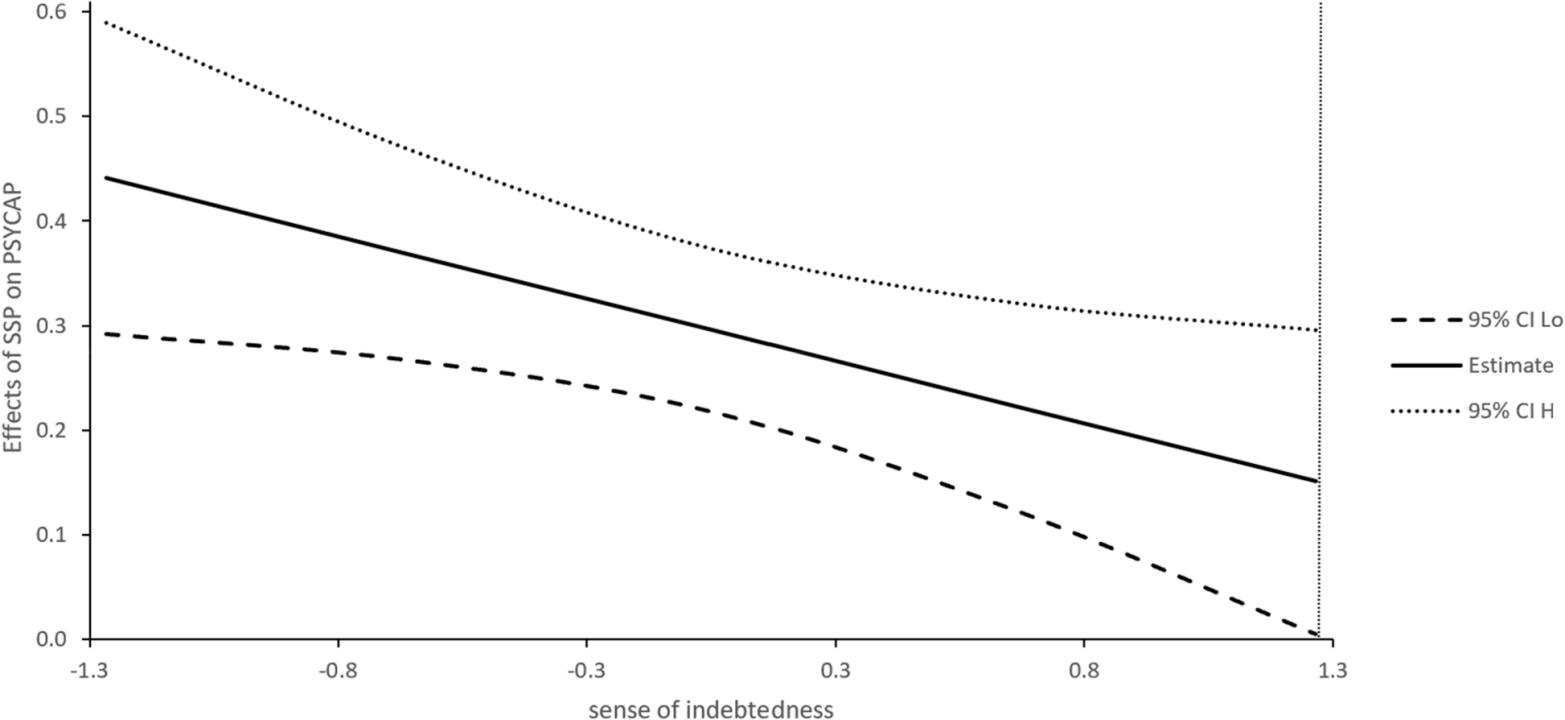
Effect of SSP on PSYCAP conditional on INDEB

**Fig. 2:**
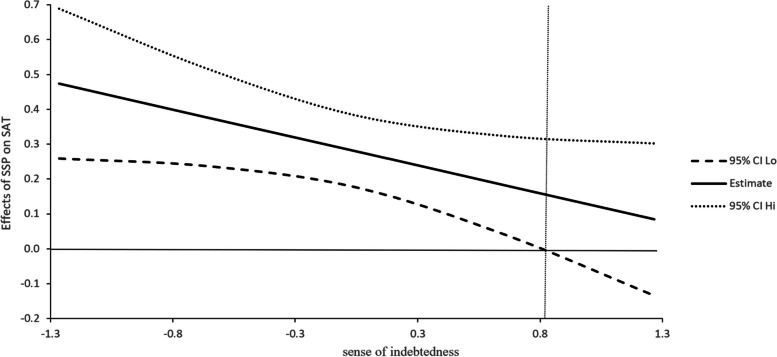
Effect of SSP on LS conditional on INDEB

**Fig. 3:**
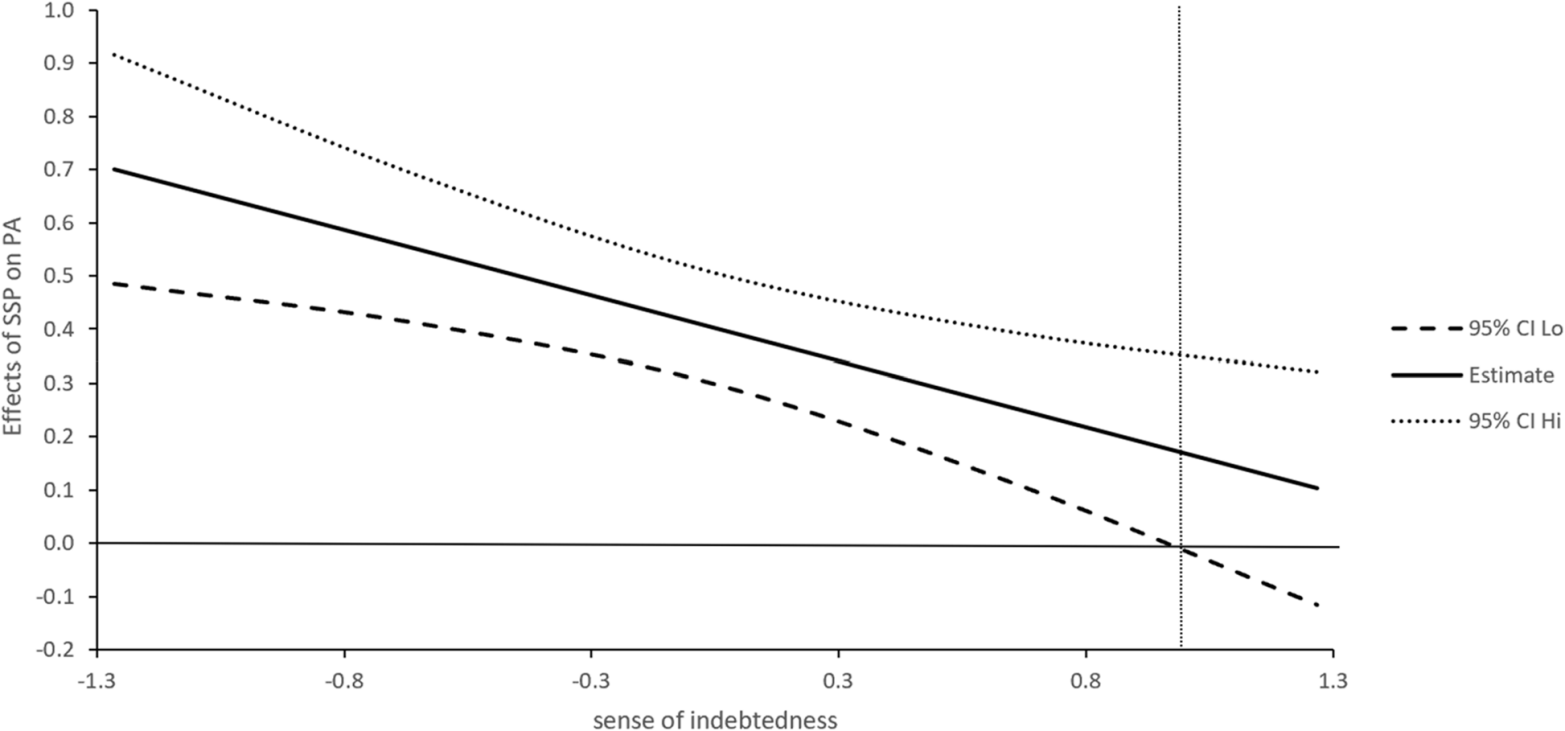
Effect of SSP on PA conditional on INDEB

The J-N technique is used to determine the interval of the moderated effect of sense of indebtedness. Figure 1 shows that when the value of sense of indebtedness is lower than 1.2 standard deviations above the mean, the relationship between social support provision and psychological capital is significant; Fig. 2 shows that when the value of sense of indebtedness is lower than 0.82 standard deviations above the mean, social support provision has a significant relationship with life satisfaction; Fig. 3 shows that when the value of sense of indebtedness is lower than 0.95 standard deviations above the mean, social support provision has a significant relationship with positive affect. Overall, with the increase in the sense of indebtedness, the relationships between social support provision and outcome variables are weakening.

## Discussion

The results confirmed a significant association between social support provision and psychological capital and subjective well-being, and the sense of indebtedness limits the relation.

### Social support provision and subjective well-being

Numerous studies have confirmed the positive predictive effect of social support on subjective well-being. This study revealed that social support provision could be significantly associated with subjective well-being. The results verified the self-determination theory and positive activities model [30], consistent with the studies about the psychological benefits of prosocial behaviors such as volunteering and donating [58, 59]. College students with low socioeconomic status provide emotional support to others rather than monetary and instrumental support, which also benefits their subjective well-being. Additionally, the study confirmed the model of longitudinal well-being [24]. This study indicated that social support provision, as an intentional positive act on relationships, can engender the provider's well-being. This reminds us that providing resources for individuals is not the only choice to improve individuals’ subjective well-being. We can try to guide individuals to actively construct their well-being, such as providing emotional support to others.

### The social support provision and psychological capital

The personal well-being model suggests that giving behavior has the function of enhancing psychological resources [60], even kindness meditation can build personal resources [61]. This study confirmed that giving emotional support to others to help them cope with their difficulties can provide experience in dealing with stressful events and improve self-competence and resilience. These acts can build a robust social support system to cope with potential difficulties so individuals can be more optimistic. In short, social support provision is significantly associated with individuals’ psychological capital.

### The moderated effect of indebtedness

This study found that providing emotional support to others by college students with low socioeconomic status benefits the construction of individuals’ psychological resources and the acquisition of subjective well-being. However, there is a specific restriction on the positive effect of social support provision. Impoverished college students have a sense of indebtedness after receiving the state’s aid, which induces repayment-style giving, which is forced giving essentially, causing the benefits of social support provision to reduce, validating the self-determination theory [62]. Involuntary behavior does not positively impact the actor [63] and even negatively impacts the perpetrator due to detriment to autonomy [38, 64, 65]. The results are consistent with Inagaki’s hypothesis that social support provision becomes a burden when it lacks autonomy, and the benefits would be reduced. On the contrary, if the provision satisfies the individual’s autonomy needs, it can play a positive role.

### Limitations and implications

First, the study adopted a cross-sectional design to explore the influence of social support provision on psychological capital and subjective well-being. However, the experimental design can lead to more convincing conclusions [66], and future research should adopt an experimental design. Second, the data collection for this research was done using a self-report method, which entails a certain degree of moral evaluation in providing social support to others. It may overestimate the social support given by oneself. Using a combination of self-reports and field experiments to measure the social support provision by individuals may more accurately decrease the influence of socially desirable responses. Third, the test and control for common method variance may not be sufficient. In terms of procedure remedy, the counterbalancing question order is only implemented at the school level and not at the participants' level, and anonymization is used to eliminate the evaluation apprehension of the participants, but the effect has not been thoroughly evaluated; In terms of statistical remedy, CFA Marker Technique needs to set a marker in the questionnaire, the study did not set markers in advance, so the technique has not been implemented. Fourth, take undergraduates who receive national financial aid as research subjects, and their sense of indebtedness is generated by receiving financial support. However, this study does not consider other types of support, such as emotional support, in which the sense of indebtedness is not as strong as receiving financial support. Therefore, future research should determine the cause of indebtedness to examine its influence on the relationship between social support provision and subjective well-being.

Despite the shortcomings mentioned above, the results of this study have specific implications. From a theoretical perspective, this study focuses on the benefit of social support provision, which expands the field of social support research and verifies the hypothesis that social support provision plays a positive role in individuals’ development, such as psychological capital and well-being; from a practical perspective, social support provision can improve psychological capital and subjective well-being. Therefore, it is not enough to provide social support to improve mental health and promote the subjective well-being of marginalized groups. One should also guide them to contribute to others and society. In addition, the positive effect of support provision is restricted by the individual’s sense of indebtedness, so when guiding individuals to provide social support, they should be given freedom, and it should satisfy their need for autonomy.

## Conclusion

The study explores the relationship between social support provision, sense of indebtedness and psychological capital, and subjective well-being. The results prove that social support provision is significantly related to subjective well-being and psychological capital. The association is affected by the individual’s sense of indebtedness; college students with low sense of indebtedness can benefit more from social support provision.

## Data Availability

The datasets used and analyzed during the current study are available from the corresponding author on reasonable request.

## Abbreviations

SSP: Social Support Provision
PSYCAP: Psychological Capital
LS: Life Satisfaction
PA: Positive Affect
NA: Negative Affect
INDEB: Sense of Indebtedness
